# PepLM-GNN: A graph neural network framework leveraging pre-trained language models for peptide-protein binding prediction

**DOI:** 10.1371/journal.pcbi.1014084

**Published:** 2026-03-24

**Authors:** Ke Yan, Meijing Li, Shutao Chen, Tianyi Liu, Jing Hao, Bin Liu, Zhen Li

**Affiliations:** 1 School of Computer Science and Technology, Beijing Institute of Technology, Beijing, China; 2 Zhongguancun Academy, Beijing, China; 3 SMBU-MSU-BIT Joint Laboratory on Bioinformatics and Engineering Biology, Shenzhen MSU-BIT University, Shenzhen, Guangdong, China; Shiraz University, IRAN, ISLAMIC REPUBLIC OF

## Abstract

**Motivation:**

The precise prediction of peptide-protein interaction (PepPI) is a core support for promoting breakthroughs in peptide drug research, as well as understanding the regulatory mechanisms of biomolecules. Researchers have developed several computational methods to predict PepPI. However, existing computational methods also have significant limitations. At the level of data feature characterisation, the problem of PepPI does not conform to the Euclidean axioms, making it difficult for conventional prediction methods to effectively measure the underlying correlations between peptides and proteins. At the level of model generalisation performance, existing approaches are often hampered by insufficient generalisation ability, as manifested by their markedly degraded performance in cold start scenarios involving novel peptides, novel proteins, and novel binding pairs.

**Results:**

In this study, we propose a computing framework, PepLM-GNN, that integrates a pre-trained language ProtT5 model with a hybrid graph network for accurate identification of PepPI. This model constructs a graph by using ProtT5-extracted semantic context features of peptides and proteins to form heterogeneous nodes, with edges connecting interacting peptide-protein pairs. The hybrid graph network Graph Convolutional Networks (GCN) provides the comprehensive information of the peptide and protein sequences, while employing the Graph Isomorphism Network (GIN) to capture the global interactions between them. Specifically, the GCN aggregates both the semantic context information of node sequences and local neighbourhood information, effectively representing non-Euclidean data. To capture the global associations, we adopt a GIN strategy to optimize the cross-node feature interaction and transfer process, thereby enhancing the generalisation performance of addressing the cold start scenario. Compared with the existing advanced methods, PepLM-GNN demonstrated highly accurate performance and robustness in predicting the PepPI. We further demonstrated the capabilities of PepLM-GNN in virtual peptide drug screening, which is expected to facilitate the discovery of peptide drugs and the elucidation of protein functions.

## Author summary

We propose a computational framework, PepLM-GNN, that integrates the ProtT5 pre-trained language model with a hybrid graph network. Specifically, the semantic features of peptides and proteins are extracted using ProtT5 to construct a graph. Within the hybrid graph network, GCN model aggregates semantic and local neighborhood information from node sequences, enabling an adequate representation of non-Euclidean data. Meanwhile, GIN model is utilized to optimize the process of cross-node feature interaction and transmission, thereby enhancing the generalization performance in addressing cold-start scenarios. Experimental results demonstrate that PepLM-GNN outperforms existing state-of-the-art methods in both accuracy and robustness for PepPI prediction. Moreover, PepLM-GNN can be applied to virtual peptide drug screening, thereby accelerating the development of peptide drugs. Furthermore, we have established a public online service platform (http://bliulab.net/PepLM-GNN) to facilitate the practical application.

## 1. Introduction

The peptide-protein interaction (PepPI) is widely present in living organisms and has been a core issue in the field of life sciences. It plays a crucial role in numerous key physiological processes, including cell signal transduction, metabolic regulation, and so on [1–3]. For instance, in the cellular signaling pathway, peptide hormones (such as insulin) can specifically bind to protein receptors on the cell surface, thereby triggering a series of cascade reactions and achieving precise regulation of biological blood glucose levels [4]. The peptide-protein complexes on the surface of antigen-presenting cells in the immune response can activate T cells to resist the invasion of pathogens [5–7].

Clarifying the PepPI is of great significance in many fields. In the process of drug development, it can help researchers clarify their goals and promote the emergence of new drugs [8–14]. Take cancer treatment as an example. Scientists can develop new anti-cancer drugs based on the abnormal PepPI within tumour cells [15]. These drugs have fewer side effects and more effective therapeutic benefits. In the field of disease mechanism research, elucidating the molecular mechanisms of the PepPI can reveal the root causes of diseases and open up new paths for their early diagnosis and effective treatment. For instance, in the study of neurodegenerative diseases, abnormal PepPI may provide new insights for combating these diseases [16].

In traditional research, PepPI have been determined through experimental methods such as yeast two-hybrid, co-immunoprecipitation, and surface plasmon resonance. However, these experimental methods differ in terms of cost, time, and throughput [2]. With the rapid update of deep learning technology, its powerful data-driven feature learning capabilities have provided certain solutions for predicting PepPI [17–19]. Compared to traditional machine learning methods that rely on manual feature engineering, deep learning models can automatically extract complex, nonlinear patterns from massive biological sequences and structural data, thereby significantly reducing the subjectivity and complexity of manual feature screening [14,20–28]. This advantage has been fully confirmed in fields such as natural language processing and image recognition [29,30]. In the PepPI study, deep learning has demonstrated great potential in uncovering hidden molecular associations, providing a novel strategy for analysing complex biological interaction mechanisms [31–34].

Among the existing deep learning-driven prediction methods for biomolecular interactions, the representative methods for PepPI prediction include CAMP [35] and IIDL-PepPI [36]. CAMP utilized the multi-channel sequence information and the conventional neural networks framework to predict the interactive and binding residues [35]. IIDL-PepPI constructed a bidirectional attention module to represent the contextual information of peptides and proteins, achieving pragmatic analysis of protein language. Meanwhile, it adopts a progressive transfer learning framework to address the peptide-protein interaction problem [36]. In addition, representative methods in the fields of protein-protein interaction (PPI) and drug-target interaction prediction also serve as references for related research. The HIGH-PPI constructed a two-view graph network to obtain the cross-protein global interaction and the local residue association within protein-protein interactions, respectively [37]. DrugBAN utilized a domain-adapted deep bilinear attention network framework to predict the local interactions of drug-target pairs [38].

However, these computational methods of PepPI prediction still have several challenges. Firstly, PepPI data belong to non-Euclidean data, where the peptide-protein interaction presents complex network topological characteristics. For instance, the same protein interacts with multiple peptides, forming distinct binding sites for each peptide. Relying solely on hand-crafted features is insufficient to capture these complex bindings explicitly. Secondly, the existing methods lie in their limited generalisation capability, especially for the cold start scenario of “novel peptides/novel proteins/novel binding pairs”.

In this study, we propose an accurate and interpretable framework, PepLM-GNN, that integrates a pre-trained language ProtT5 model and hybrid graph neural networks. The core innovation lies in optimizing PepPI modelling through the collaborative design of GCN and GIN. The nodes in the graph network are the semantic context features of peptides and proteins, while the edges are their interaction relationship. GCN extracts the local-level information via the feature associations between nodes and their direct neighbors. GIN is used to capture the global-level topological structure through the equivalence class discrimination mechanism. Specifically, the contributions of this article include:

(1) Our model employs GCN’s local neighbourhood aggregation to extract feature associations between nodes and their direct neighbours effectively. This process provides detailed support for micro-interaction in the model and meeting the non-Euclidean data modelling requirements of PepPI.
(2) To mitigate the limitations of GCN (e.g., gradient dispersion and over-smoothing that lead to the feature discriminability), we introduce GIN to enhance feature discriminability and capture the global topological structure. The hybrid framework enhances the generalisation ability and adaptability to the cold start scenario, including novel peptides, novel proteins, and novel binding pairs.
(3) An interpretable interaction subgraph is constructed by extracting important associations from the PepLM-GNN. The biological significance of the interpretable components derived from the subgraph is evaluated through a functional enrichment analysis experiment. Moreover, PepLM-GNN has been extended for applications in the alanine scanning of peptides, demonstrating a probability-driven approach to peptide drugs.
(4) To facilitate the use of researchers, we have built an online web server based on the proposed model, which can be accessed at http://bliulab.net/PepLM-GNN.

## 2. Results and discussion

### 2.1 Comparison with the other baseline methods on the benchmark dataset

In this section, we compared the model’s performance with the other baseline methods on the benchmark dataset. The compared baseline methods include traditional machine learning models (LR [39], RF [40], SVM [41,42]) and classic deep learning models (CAMP [35], DrugBAN [38], IIDL-PepPI [36], HIGH-PPI [37]). The performance of the models is evaluated using four metrics: AUC, AUPR, F1, and ACC.

**Fig 1:**
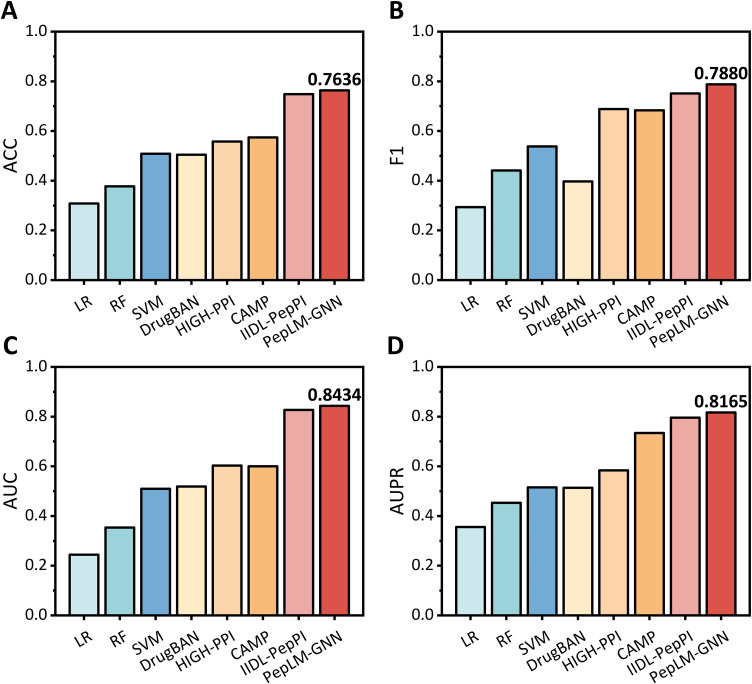
Performance of PepLM-GNN against other baseline methods on the benchmark dataset via five-fold cross-validation. The benchmark dataset (total 17244 samples) is derived from peptide-protein complex structures in the RCSB PDB database (before October 2022), containing 8622 positive samples (interacting peptide-protein pairs) and 8622 negative samples (non-interacting pairs) after data filtering. All reported performance metrics (e.g., ACC, AUC) represent the average values obtained from the five-fold cross-validation conducted on the benchmark dataset.

As shown in **Fig 1** and S1 Table, the PepLM-GNN demonstrates advantages in the binary PepPI prediction task. Its AUC of 0.8434 is superior to baseline methods, demonstrating the proposed method’s excellent ability to distinguish PepPI. Compared with traditional machine learning methods that relied solely on hand-crafted features, the proposed method utilized the pre-trained ProtT5 [43] language model as a sequence encoder. This approach provides a more comprehensive and semantically rich representation of biological sequences, thereby significantly improving the model’s expressive power and predictive accuracy. Compared with the IIDL-PepPI [36] that utilized the pre-trained ProtBert [44] model and progress transfer learning, the proposed method utilized the GCN framework to capture the non-Euclidean data space of PepPI. By propagating and aggregating feature information across neighbouring nodes, the proposed method effectively learns from the local interaction context. Although methods such as DrugBAN [38] and HIGH-PPI [37] utilized network topological structures, they lack high-quality sequence semantic priors and have relatively low prediction performance. Therefore, PepLM-GNN achieves superior performance in identifying PepPI and maintains stable performance compared to other baseline methods.

To further verify the statistical significance of the performance superiority of PepLM-GNN, we conducted statistical t-tests on the mean ACC values of PepLM-GNN and all comparative baseline methods derived from five-fold cross-validation on the benchmark dataset, and the specific *p*-values are summarized in **Table 1**. As shown in **Table 1**, all *p*-values of the statistical t-tests between PepLM-GNN and other baseline methods are less than 0.05, which quantitatively demonstrates that the PepLM-GNN is statistically significantly higher than the comparative methods, and the performance advantage of our model on the benchmark dataset is not caused by random factors.

**Table 1:** Statistical t-test *p*-values for ACC of comparative methods versus PepLM-GNN. This table reports the *p*-values of statistical t-tests between PepLM-GNN and other baseline methods, based on the mean ACC values from five-fold cross-validation on the benchmark dataset (17244 samples: 8622 positive and 8622 negative pairs). A *p*-value < 0.05 indicates that the PepLM-GNN is statistically significantly higher than that of the comparative method.

Method	LR	RF	SVM	CAMP	DrugBAN	IIDL-PepPI	HIGH-PPI
***p*-value**	8.07 × 10^-8^	1.55 × 10^-7^	8.18 × 10^-7^	2.67 × 10^-6^	7.67 × 10^-7^	2.83 × 10^-2^	1.93 × 10^-6^

### 2.2 Comparison with other baseline methods on independent test and cold start test datasets

**Fig 2:**
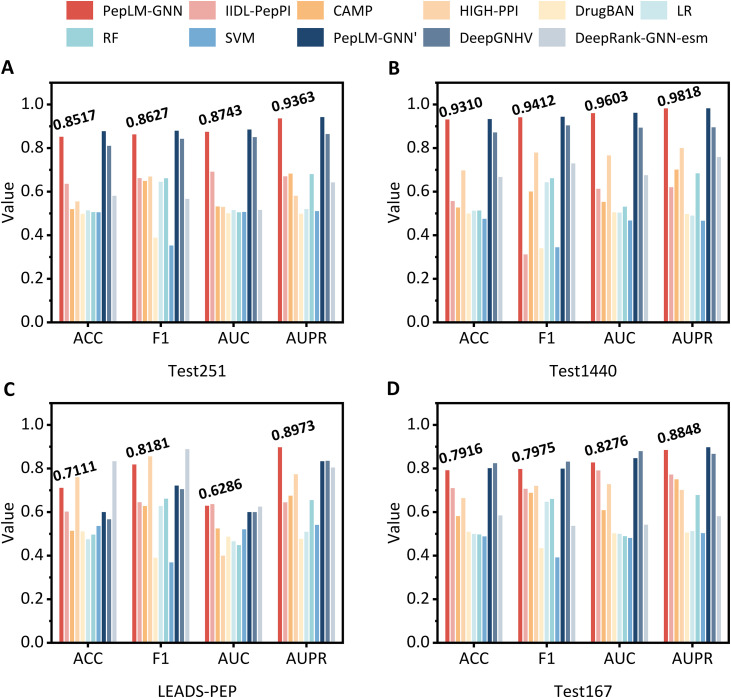
Performance of PepLM-GNN against other baseline methods on four independent test datasets. The four test datasets include: Test1440 (1440 positive peptide-protein pairs and 1440 negative pairs, sourced from the RCSB PDB database, January 2023-July 2024), LEADS-PEP (52 positive pairs and 52 negative pairs, a classic benchmark for evaluating peptide-protein docking performance), Test251 (249 positive pairs and 249 negative pairs), and Test167 (255 positive pairs and 255 negative pairs, derived from the RCSB PDB database, October-December 2022). PepLM-GNN’, DeepGNHV, Deep-GNN-esm are only applied to test subsets with available structural data. All performance metrics represent the mean values averaged across the five models derived from five-fold cross-validation on each independent test set.

To verify the model’s generalisation ability, we conduct comparative experiments on four independent test datasets, including LEADS-PEP, Test167, Test251, and Test1440. The compared baseline methods contain traditional machine learning methods (SVM, RF, LR) and deep learning methods (CAMP [35], HIGH-PPI [37], DrugBAN [38], IIDL-PepPI [36], DeepRank-GNN-esm [45], DeepGNHV [46]). The hyperparameters of all baseline methods were optimized to their optimal values, ensuring a fair comparison. We evaluated the performance in terms of AUC, AUPR, F1, and ACC on the four independent test datasets, and the results are illustrated in **Fig 2**.

As shown in **Fig 2**, the results indicate that PepLM-GNN outperforms other state-of-the-art methods on binary PepPI prediction. The limited generalization of baseline methods, such as the CAMP [35], makes it challenging to capture the fundational sequence semantic knowledge, which is critical for predicting unseen PepPI. In contrast, the proposed method utilizes the pre-trained ProtT5 [43] model, which was constructed based on the large-scale unsupervised protein sequence, to provide rich semantic information. Moreover, the proposed method utilizes the GIN to capture the global topological association between the peptide and protein. By learning these inherent relational associations, PepLM-GNN has a generalizable model of interaction information, thereby improving its generalization capability for accurate binding prediction on unseen PepPI.

To confirm the statistical significance of the performance advantage of PepLM-GNN on independent test data, we further conducted statistical t-tests on the mean ACC values. The test data was derived from the union of four independent test datasets, and the mean ACC values were calculated via five-fold cross-validation. The specific *p*-values of the t-tests are summarized in **Table 2**. It can be seen from **Table 2** that all *p*-values of the statistical t-tests between PepLM-GNN and the comparative methods were less than 0.05, indicating that the superior performance of PepLM-GNN on the integrated test set is statistically significant, and this advantage is stable and reliable.

**Table 2:** Statistical t-test *p*-values for ACC between comparative methods and PepLM-GNN across four combined test sets. This table reports the *p*-values of statistical t-tests between PepLM-GNN and other comparative methods. The test data is the union of four independent test datasets. T-tests are based on the mean ACC values from five-fold cross-validation across five folds on the combined dataset. A *p*-value < 0.05 indicates that PepLM-GNN is statistically superior.

(a) First part of comparative methods					
Method	LR	RF	SVM	CAMP	DrugBAN
*p*-value	6.34 × 10^-6^	6.29 × 10^-6^	4.37 × 10^-6^	1.71 × 10^-5^	6.07 × 10^-6^
**(b) Second part of comparative methods**					
Method	IIDL-PepPI	HIGH-PPI	DeepRank-GNN-esm	DeepGNHV	
*p*-value	1.10 × 10^-3^	2.01 × 10^-5^	5.72 × 10^-6^	1.88 × 10^-2^	

**Fig 3:**
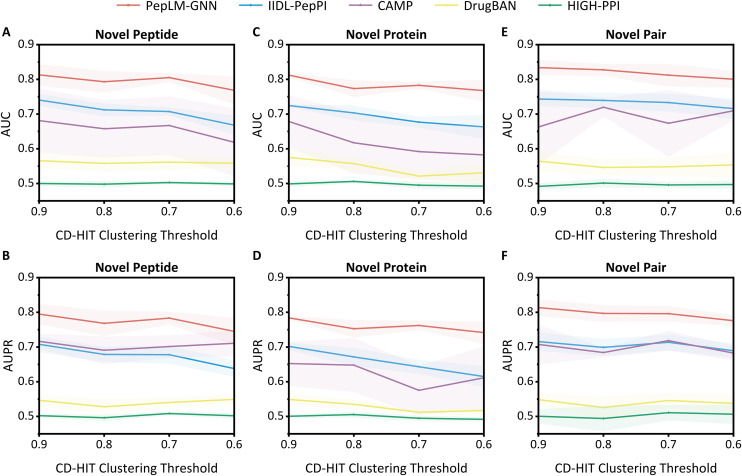
Comparison of PepPI predictions based on cluster-split datasets for predicting novel peptides, proteins, and peptide-protein pairs. Error bars represent the mean ± standard deviation of cross-validation experiments. The cluster-split (cold start) dataset is constructed using the CD-HIT clustering algorithm with four thresholds (0.6, 0.7, 0.8, 0.9), following the CAMP strategy: no entities from the same cluster appear in both training and test sets, resulting in three sub-datasets (“novel peptides”, “novel proteins”, “novel binding pairs”). All performance metrics are the mean ± standard deviation of five folds from five-fold cross-validation.

Furthermore, to systematically evaluate the generalisation ability of the PepLM-GNN, we utilize the CD-HIT algorithm to construct cold-start test datasets comprising novel peptides, novel proteins, and novel protein-peptide pairs. PepLM-GNN was evaluated against other advanced deep learning methods using AUC and AUPR, and the results are shown in **Fig 3**.

The results showed that (1) PepLM-GNN outperformed compared methods, including IIDL-PepPI [36], CAMP [35], DrugBAN [38], and HIGH-PPI [37] in the four clustering threshold scenarios. (2) When the clustering threshold decreases and the sequence similarity between the training set and the test set weakens, the decline in the model’s performance is relatively small, demonstrating a robust generalisation ability. By leveraging its equivalence class discrimination mechanism,the GIN model in the proposed method accurately capture the global topological structure of molecular interactions. When the sequence similarity decreases, cross-sample association can still be achieved through the consistency of structural patterns, thereby enhancing the model’s generalization ability. Therefore, PepLM-GNN demonstrates superior and more robust performance compared to the baseline methods on the independent test and cold start test datasets.

### 2.3 Comparative performance of PepLM-GNN with existing pre-trained language models

In this section, we evaluated four pre-trained language models [47] on the benchmark dataset to assess the impact of different feature encoding strategies on the PepPI prediction. The four embedding features generated by pre-trained language models are TAPE [48], ProtBert [44], ESM-2 [49], ESM-3 [50], and the proposed method based on ProtT5 [43]. TAPE [48] is mainly used for modelling the context of biological sequences, ProtBert [44] focuses on general semantic representation, and ESM-2 [49] integrates evolutionary information. As shown in **Table 3**, the PepLM-GNN model exhibits outstanding predictive performance across all four metrics. Unlike the biological language model (e.g., TAPE [48] and ProtBert [44]), the ProtT5 [43] can capture the subtle differences in key functional motifs of short peptide sequences and model the context of large-scale corpora [51]. By leveraging adaptive sequence embedding and hybrid graph networks, PepLM-GNN can effectively predict the interaction of peptide-protein pairs.

**Table 3:** Performance comparison between PepLM-GNN and other pre-trained language models on the benchmark dataset in terms of ACC, F1, AUC, and AUPR. The benchmark dataset contains 17,244 samples (8,622 positive and 8,622 negative pairs). All metrics (ACC, F1, AUC, AUPR) are the mean ± standard deviation of five folds from five-fold cross-validation.

Method	ACC	F1	AUC	AUPR
ProtBert [44]	0.6620 _± 0.0154_	0.7299 _± 0.0061_	0.7565 _± 0.0087_	0.7357 _± 0.0127_
TAPE [48]	0.6850 _± 0.0111_	0.7427 _± 0.0042_	0.7753 _± 0.0095_	0.7533 _± 0.0098_
ESM-2 [49]	0.6924 _± 0.0065_	0.7440 _± 0.0042_	0.7864 _± 0.0061_	0.7653 _± 0.0078_
ESM-3 [50]	0.7013 _± 0.0068_	0.7500 _± 0.0034_	0.7950 _± 0.0032_	0.7690 _± 0.0052_
**PepLM-GNN**	**0.7636** _**± 0.0117**_	**0.7880** _**± 0.0034**_	**0.8434** _**± 0.0060**_	**0.8165** _**± 0.0103**_

**Note:** The specific versions/parameter configurations of the pre-trained language models used in this study are as follows: ProtT5: ProtT5-XL-UniRef50; ESM-2: esm2_t33_650M_UR50D; ESM-3: esmc-300m-2024-12; ProtBert: ProtBert-BFD; TAPE: ProteinBertModel (bert-base). All models adopt the default parameter settings consistent with their official pre-trained versions and original published papers.

**Table 4** shows that the *p*-values of the t-tests between the PepLM-GNN and all variant models are less than 0.05, indicating that the ACC of the PepLM-GNN using ProtT5 is statistically significantly higher than that of the variants with other pre-trained models substituted. This confirms that ProtT5 is more suitable for the PepPI prediction task in our model framework and can provide more effective feature representation for subsequent graph neural network processing.

**Table 4:** *P*-values of statistical t-test for ACC: PepLM-GNN with different pre-trained model on five-fold cross-validation set. This table reports the *p*-values of statistical t-tests between the PepLM-GNN (using ProtT5) and other pre-trained language models. T-tests are based on the mean ACC values from five-fold cross-validation on the benchmark dataset (17244 samples: 8622 positive + 8622 negative pairs). A *p*-value < 0.05 indicates that the original PepLM-GNN has a statistically significantly higher ACC than the variant.

Method	ProtBert	TAPE	ESM-2	ESM-3
***p*-value**	3.19 × 10^-5^	8.78 × 10^-5^	1.29 × 10^-4^	2.18 × 10^-4^

### 2.4 Ablation study of PepLM-GNN on the benchmark dataset

To explore the contribution of three components of PepLM-GNN to the prediction performance, this section designs an ablation study on the benchmark set. We systematically developed several variant models by ablating each of the three individual modules. Model A only retains the ProtT5 [43] embedding and classification module, and the features obtained through ProtT5 [43] are directly used for classification. The GAT module was added to Model A to create Model B, which was then used to evaluate the impact of GAT on overall performance. The GCN module was added to Model A to create Model C, which was used to compare the effectiveness of GCN in the prediction task. The GIN module was added to Model A to create Model D, which was further used to verify the performance of GIN in the prediction task. The ablation study results for ACC and AUC are summarised in **Table 5**.

**Table 5:** Ablation experiment performance of PepLM-GNN on the benchmark set using five-fold cross-validation. Ablation variants include: A (pretrained module + classification module), B (pretrained module + GAT + classification module), C (pretrained module + GCN + classification module), D (pretrained module + GIN + classification module), and the complete PepLM-GNN model (pretrained module + GCN + GIN + classification module). All metrics (ACC, AUC) are the mean ± standard deviation across five folds of a five-fold cross-validation.

Method	GCN module	GIN module	GAT module	ACC	AUC
A	✖	✖	✖	0.7350_±0.0134_	0.8160_±0.0080_
B	✖	✖	✔	0.7351_±0.0105_	0.8223_±0.0096_
C	✔	✖	✖	0.7453_±0.0107_	0.8346_±0.0036_
D	✖	✔	✖	0.7475_±0.0235_	0.8341_±0.0149_
**PepLM-GNN**	✔	✔	✖	**0.7636**_**±0.0117**_	**0.8434**_**±0.0060**_

As shown in **Table 5**, model A retains only the pre-training and classification modules, performing the worst in terms of ACC and AUC. This model relies solely on sequence features for end-to-end classification and is unable to capture the interaction relationships between molecules, making it challenging to identify the PepPI. Compared with model A, models C and D exhibit higher performance, highlighting the unique value of graph networks. GCN enhances the structural connectivity of nodes by aggregating local neighbourhood features, while GIN enhances the perception of the global topological structure through iterative updates. At the same time, it can alleviate the problems of gradient dispersion and over-smoothing that are prone to occur in the deep training of GCN. These two mechanisms address the information deficiency of single-sequence features from both local and global perspectives. The research model PepLM-GNN combines the fine-grained local-structure modelling of GCN with the in-depth exploration of global topology by GIN, forming a functional synergy. Therefore, the ablation studies demonstrated that integrating the interaction network topology with sequence semantics information is essential for achieving effective prediction in the PepPI problem.

To further quantitatively verify the significant contribution of each core module to the model’s performance, we conducted t-tests on the mean ACC values of the full PepLM-GNN model and each ablation variant (based on five-fold cross-validation on the benchmark dataset), and the corresponding *p*-values are reported in **Table 6**. It can be observed from **Table 6** that all *p*-values of the statistical t-tests between the complete model and each ablation variant are less than 0.05, which statistically confirms that removing any core module will lead to a significant decrease in the ACC of the model. This fully demonstrates that each core module of PepLM-GNN (GCN, GIN, etc.) makes a statistically significant contribution to the model’s PepPI prediction performance, and the model’s hybrid architecture design is scientifically sound and reasonable.

**Table 6:** *P*-values of statistical t-test for ACC: Ablation experiments of PepLM-GNN on five-fold cross-validation set. This table reports the *p*-values from t-tests comparing the complete PepLM-GNN model with each of its ablation variants. Ablation variants include: A (pretrained module + classification module), B (pretrained module + GAT + classification module), C (pretrained module + GCN + classification module), D (pretrained module + GIN + classification module). T-tests are based on the mean ACC values from five-fold cross-validation on the benchmark dataset (17244 samples: 8622 positive + 8622 negative pairs). A *p*-value < 0.05 indicates that the removed module makes a statistically significant contribution to the ACC of PepLM-GNN.

Method	A	B	C	D
***p*-value**	4.01 × 10^-3^	4.06 × 10^-3^	1.75 × 10^-2^	2.54 × 10^-2^

### 2.5 The application of PepLM-GNN in extended tasks

To explore the interpretability and biological significance of PepPI prediction, we conducted multi-level experiments, including the key residue identification and interaction network analysis, and gene enrichment analysis.

#### 2.5.1 Virtual screening of key residues involved in the interaction.

To verify the practicality of PepLM-GNN, we combined computer simulation and experimental data to compare the model prediction results with the biometric results and verify its consistency in identifying key amino acids. We conducted alanine-scanning virtual screening of the SALL4 (1–12) peptide segment [52]. Replace each amino acid in this peptide segment with alanine in sequence to construct a series of mutants. Using the established model, predict the binding probability of each mutant to RBBp4, respectively. To quantify the impact of alanine substitution on the peptide-protein binding ability, the relative change in binding likelihood can be expressed as (binding probability of wild-type SALL4 (1–12) peptide-binding probability of alanine mutant)/binding probability of wild-type SALL4 (1–12) peptide [36].

**Fig 4:**
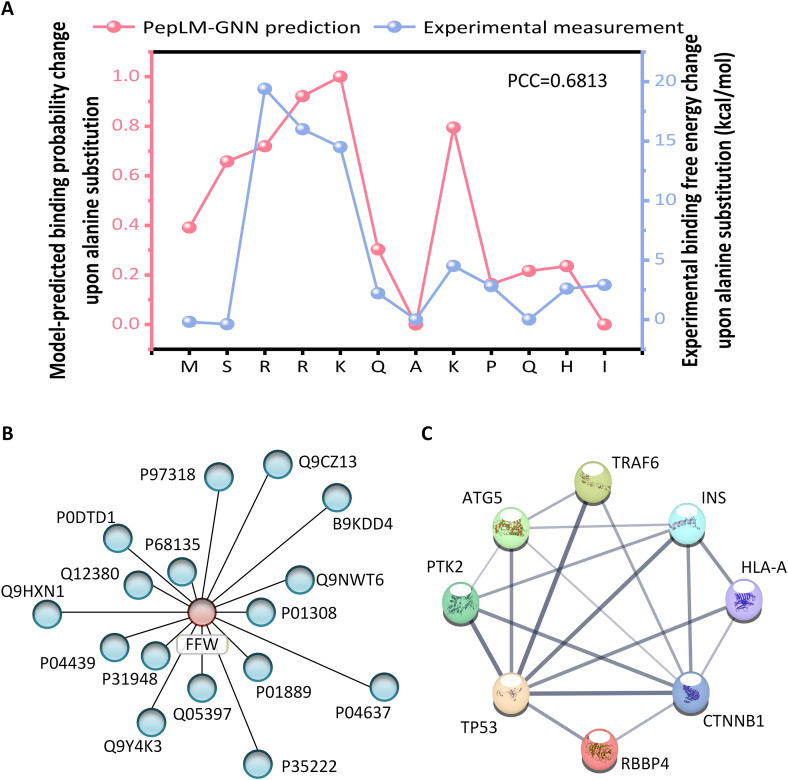
Application of PepLM-GNN in virtual peptide drug screening. **(A)** Relative changes in the predicted binding probability of RBBp4 and SALL4 peptide alanine mutants by PepLM-GNN compared with experimentally measured changes in binding free energy. **(B)** Protein subgraphs interacting with FFW peptides extracted using the GNNExplainer tool. **(C)** Functional enrichment analysis of proteins interacting with the FFW peptide.

The model predicts that the relative changes in the binding probabilities of alanine mutants corresponding to the peptide “RRK” sequence (arginine R, lysine K) are all very high. Compared with the experimental data of the binding free energy measured in the SALL4 study [52], as shown in **Fig 4A**, the trends of the two are highly consistent. Among the three residue positions with the most significant changes in binding free energy measured experimentally, two correspond to the “RRK” sequence mutants, highlighting the core role of this sequence in the interaction between SALL4 and RBBp4. This finding is consistent with the conclusion of “Arg3, Arg4, Lys5 as key interaction residues” in the SALL4 study, which verifies the consistency between model predictions and experimental measurements in identifying key amino acids. This high consistency between prediction and experiment stems from the in-depth analysis of PepPI by the model architecture. The ProtT5 [43] pre-trained model mined the semantic information of sequences such as “RRK”, providing biological semantic support for identifying key residues. The graph network framework can precisely model the local residue interactions at the binding interface, perceive the global topological structure, and accurately capture the microscopic interactions between the “RRK” and RBBp4 binding sites. The synergy effect of this model architecture enables it to identify functional key regions at the sequence semantic level and analyse the specific contributions of residues at the network topology level, achieving precise identification of key binding residues.

#### 2.5.2 Subgraph identification of FFW interacting proteins.

To study the interpretability and biological significance of the model’s predictions, we used the target peptide FFW as an example for subgraph analysis and functional verification. FFW is an HCC therapeutic peptide developed based on the SALL4 key sequence, and its functional core lies in the specific interaction with RBBp4. We analysed the proposed model using the GNNExplainer tool [53], extracted the protein subplots that might interact with the target peptide FFW, and presented the core proteins with the highest rankings. **Fig 4B** shows that these proteins form functional modules through close physical interactions, suggesting that they may jointly participate in the signalling pathways related to liver cancer development.

#### 2.5.3 Functional enrichment and clinical significance validation of FFW interacting proteins.

This section performs a functional enrichment analysis on the aforementioned core proteins [36]. **Fig 4C** shows that they are significantly enriched in liver cancer-related pathways (enrichment *p*-value 3.63 × 10 ⁻ ²). This not only verified the accuracy of the model in identifying key interaction pairs (FFW-RBBp4) but also directly confirmed the mechanism by which “RRK residues regulate tumorigenesis by targeting RBBp4”, which is consistent with the core role of the “RRK” sequence in SALL4. From the virtual screening of key residues in SALL4 to the validation of FFW interaction networks and pathways, the proposed model, PepLM-GNN, demonstrates stable interpretability and biological consistency in PepPI prediction.

## 3. Conclusion

This study proposes a PepLM-GNN method that integrates a pre-trained ProtT5 model and hybrid graph neural networks for the PepPI prediction. The model employs a GCN to extract the local neighbourhood features of molecules, enabling the capture of short-range interactions and precise modelling of non-Euclidean structures. Furthermore, GIN relies on the equivalence class discrimination mechanism to capture the global node interaction pattern, thereby alleviating the gradient dispersion and over-smoothing problems in deep training of GCN and improving generalisation performance. Compared to the other state-of-the-art methods, PepLM-GNN has higher prediction accuracy and interpretability. Besides, the application of PepLM-GNN in various extended tasks highlights the framework’s versatility and potential to facilitate broader protein-related research. This achievement can be applied in areas such as the development of protein drugs, efficacy evaluation, and optimization of treatment strategies, thereby accelerating the drug research and development process. Additionally, a web server has been established and is accessible at http://bliulab.net/PepLM-GNN.

It should be noted that PepLM-GNN still has certain limitations. There are potential biases in dataset construction: although the existing benchmark dataset has undergone rigorous screening, it still falls short of fully covering protein-peptide pairs across different species and functional types, which may compromise the model’s prediction performance on rare or specialized samples. In addition, the graph construction step incurs a certain computational cost: the process of constructing molecular graphs based on sequence features consumes considerable computational resources, making it difficult to adapt to the rapid deployment requirements of low-configuration computing environments.

## 4. Materials and methods

### 4.1 Benchmark, independent test, and cold start test datasets

In this study, we construct three types of datasets to evaluate the performance of the PepPI prediction model, namely the benchmark dataset, four independent test datasets, and a cold start test dataset.

Benchmark dataset: The benchmark dataset used in this study is derived from the peptide-protein complex structures in the RCSB PDB database [54] before October 2022. The determination of residue pairs is based on the distance threshold method between α-carbon atoms ($C_{\alpha }$). When the $C_{\alpha }$ atom distance between the ligand peptide and the target protein residue is less than 5 Å, it is defined as an interacting residue pair. For amino acid residues lacking $C_{\alpha }$ atoms, the nearest inter-atomic distance is used for judgment. If the structural coordinates are incomplete or the resolution is low, making accurate judgment impossible, they are uniformly regarded as non-binding residue pairs. Then we excluded peptide-protein pairs where the proportion of unknown or non-standard amino acids exceeded 20%. After the above processing, 8622 pairs of peptide-protein interaction samples were obtained, which served as positive samples for the experiment. To construct a balanced dataset, we randomly selected an equal number of peptide-protein pairs with no interaction relationship as negative samples.

Independent test dataset: In this study, an independent test dataset, Test1440, was independently constructed. The data were sourced from peptide-protein interaction complexes in the RCSB PDB database from January 2023 to July 2024, containing a total of 1440 effective complex samples. Negative samples are generated by randomly pairing peptides with proteins to construct non-interacting pairs. In addition, we have introduced three independent test sets that have been published and widely verified in various fields. The LEADS-PEP test set [55], as a classic benchmark dataset for evaluating peptide-protein docking performance, contains a total of 52 pairs of positive and negative samples. The Test251 test set [56,57] is a commonly used independent validation dataset in the field, containing 249 pairs of positive and negative samples, and has undergone strict data screening to ensure no redundancy with the training data. The Test167 test set [36], with data derived from the peptide-protein complex in the RCSB PDB database from October to December 2022, was processed through steps such as extracting residue level labels with the PBD-BRE tool [58], filtering samples with a proportion of non-standard amino acids exceeding 20%, and excluding samples with a sequence similarity of over 80% to the training set/validation set. Ultimately, 255 pairs of positive and negative samples were retained.

Cold-start test dataset: According to the CAMP strategy [33], we independently cluster proteins and peptides using the CD-HIT algorithm (i.e., the clustering processes for the two sequence types are carried out separately, only for a single sequence type). The core data partitioning rule requires that entities from the same cluster cannot simultaneously appear in the training set and the test set, thereby ensuring the “novelty” of the cold start scenario. Based on this, we divide the benchmark data into three cold-start subsets. “New peptides” (only peptide clustering, with no overlapping peptide clusters between the training set and the test set), “new proteins” (only protein clustering, with no overlapping protein clusters between the training set and the test set), and “new binding pairs” (relying on the independent clustering results of proteins and peptides). The cold-start test dataset uses four consistent CD-HIT clustering thresholds (0.6, 0.7, 0.8, and 0.9) to construct protein and peptide sequences. It simultaneously performs independent clustering of the two sequence types under the same thresholds.

### 4.2 The architecture of the PepLM-GNN

**Fig 5:**
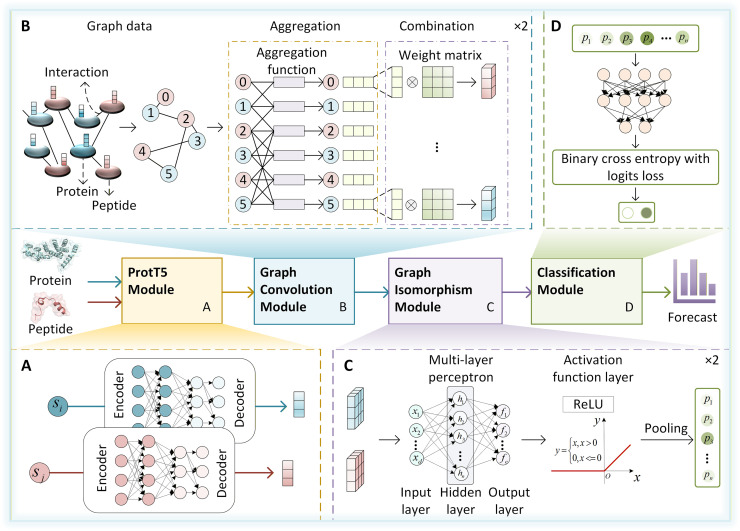
The framework of PepLM-GNN, comprising four modules: ProtT5, graph convolution, graph isomorphism, and classification.

To address the precise modelling and interpretable requirements of PepPI, this study proposes the PepLM-GNN method, a hybrid graph neural network (GNN) that achieves efficient prediction through feature learning and network topology. In the graph construct stage, a global PepPI network is established. In this network, nodes represent individual peptide or protein entities, while edges denote interaction relationships. This structure forms complex associations (e.g., a single peptide connecting to multiple target proteins, and vice versa). The hybrid graph network model adopts the collaborative architecture of GCN and GIN to achieve the local-level interaction and global-level topology information capture, respectively. GCN relies on the local neighbourhood aggregation mechanism to obtain micro-interaction details from the local topology of nodes, adapting to the non-Euclidean data characteristics of PepPI. GIN encodes the global topological dependencies of peptide-protein molecules through an equivalence class discrimination mechanism and iterative feature aggregation, while alleviating the problems of gradient dispersion and over-smoothing in the deep training of GCN. This optimizes the differentiated representation of node features, ensuring that the model can distinguish the interaction patterns of different PepPI. The model architecture is shown in **Fig 5**. Our method consists of four modules: ProtT5, graph convolution, graph isomorphism, and classification.

#### 4.2.1 ProtT5-based feature extraction.

This study constructed a sequence feature analysis architecture based on a pre-trained ProtT5 model to extract the sequence semantic context features of peptides and proteins [59]. The pre-trained ProtT5 model employs a text generation architecture and a cross-modal coding mechanism to capture the global semantic context of sequences, including their inherent long-term dependencies in sequences. When a peptide or protein sequence of length *L* is input into ProtT5, a residue-level feature matrix with dimensions of *L* × 1024 can be automatically generated. Then, we applied an average pooling operation to these residue-level features to produce a fixed-dimensional 1024-dimensional global representation vector. The calculation formula [60] is:


$$v_{\text{seq}}=\frac{\text{1}}{L}\sum_{i=\text{1}}^{L}v_{i}$$ (1)

where ${𝐯}_{i}$ is the ProtT5 feature representation. Then the ${𝐯}_{\text{seq}}$ was standardised by Z-score normalisation [61] to eliminate the influence of sequence length differences on subsequent modelling, while preserving the overall distribution of features.

During the model construction stage, the feature vectors generated by the pre-trained ProtT5 model are directly assigned as node features in the PepLM-GNN. The semantic dependencies of the sequence are captured through the cross-modal coding mechanism to provide feature input for PepPI prediction.

#### 4.2.2 Hybrid graph network framework.

This study designs a hybrid graph network framework that integrates the GCN and GIN to realise collaborative learning. The framework effectively captures the non-Euclidean data characteristics of PepPI, achieving more accurate and robust prediction performance.

The hybrid graph network framework constructs a global peptide-protein interaction network, with node 0 representing the peptide sequence and node 1 representing the protein sequence. The node features adopt the sequence-level embedding vectors extracted by the pre-trained ProtT5 model, which can avoid the defects of traditional manual features, such as “one-sided information and limited dimensions”. The edge structure is defined as a bidirectional edge index [[0,1],[1,0]], explicitly simulating the potential interaction between peptides and proteins and avoiding the ambiguity of feature transfer caused by “implicit interaction”. The feature matrix is formed by concatenating the ProtT5 embeddings of peptides and proteins, ensuring that each node initially carries complete sequence semantic information of its original sequence.

To accurately extract feature associations between nodes and their direct neighbors, and to meet the micro-modelling requirements of non-Euclidean data in PepPI, we design a GCN local aggregation module. Local neighbourhood feature learning is achieved through two layers of Graph Convolutional Neural Networks (GCNConv) [62]. The first layer maps the input features to a 64-dimensional hidden layer. Instance normalisation (IN) is employed to standardise feature distribution [63], the ReLU activation function introduces non-linearity [64], and a 20% dropout rate suppresses overfitting to avoid micro-interaction misjudgment caused by sample bias [65]. The second layer performs further neighbourhood aggregation and outputs 2 × 64-dimensional node-level local features. We strengthen the microscopic interaction between peptides and proteins, providing refined support for subsequent global modelling. The formula is as follows [66]:


$$h^{\left(l+\text{1}\right)}=\text{ReLU}\left(\text{InstanceNorm}\left({\hat{D}}^{-\text{1}/\text{2}}\hat{A}{\hat{D}}^{-\text{1}/\text{2}}h^{\left(l\right)}W^{\left(l\right)}\right)\right)$$ (2)

where $\hat{𝐀}$ is the adjacency matrix with self-loops, $\hat{𝐃}$ is the corresponding degree matrix, and ${𝐖}^{\left(l\right)}$ is the weight matrix for layer *l*.

Furthermore, the proposed method introduces a GIN-based global module to form a hybrid collaboration with GCN. This collaboration effectively mitigates issues of gradient dispersion and over-smoothing in deep GCN layers, thereby preventing the undesirable convergence of node features. In the first Graph Isomorphism Network Convolution (GINConv) layer, a multi-layer perceptron (MLP) serves to non-linearly reconstruct local features, thereby alleviating over-smoothing and enhancing the feature discriminative. In the second GINConv layer, we aggregate node features into 32-dimensional graph-level representations by using the Global Add Pooling [67]. This process captures the global topology of PepPI and significantly improves robustness in cold-start test data. The formula [68] is as follows:


$$h_{v}^{\left(l+\text{1}\right)}=f_{\Theta }\left((\text{1}+\epsilon )h_{v}^{\left(l\right)}+\sum_{u\in N(v)}h_{u}^{\left(l\right)}\right)$$ (3)

where $f_{\Theta }$ is the MLP mapping function.

#### 4.2.3 Classification module.

Based on the 32-dimensional graph-level representation output by the hybrid graph network framework, this study designs a classification module to achieve binary classification prediction of PepPI. We apply a 20% dropout rate to the graph-level representation to further reduce the model’s excessive reliance on the training data and ensure the robustness of the prediction in the cold-start test dataset. The processed features are mapped to one-dimensional logits through a linear transformation layer to reduce parameter redundancy. Use the sigmoid activation function [69] to compress logits to the interval [0,1] to obtain the interaction probability. The model formula is as follows:


PPIModel=FC(Dropout(GIN(GCN(x,E)))) (4)

where E is the edge index, and FC is the linear transformation layer.

In designing the loss function, we deeply integrate the sigmoid activation function into the calculation process of binary cross-entropy (BCE) loss [70]. It can align the prediction results with the probability attribute of PepPI interactions, thereby improving the model’s accuracy in distinguishing between interacting and non-interacting peptide-protein pairs. The formula [36] is as follows:


$$L=-\frac{\text{1}}{N}\sum_{i=\text{1}}^{N}\left(y_{i}log{\hat{y}}_{i}+(\text{1}-y_{i})log(\text{1}-{\hat{y}}_{i})\right)$$ (5)

where ${\hat{y}}_{i}$ is the predicted probability of the model, and $y_{i}$ is the true label value.

Therefore, the model PepLM-GNN we use constructs a hybrid graph neural network framework that adapts to the non-Euclidean data features of PepPI, efficiently collaborates with local and global information, and enhances the generalisation ability in cold start scenarios.

### 4.3 Training and optimization of the model

In this study, we implement the PepLM-GNN model using PyTorch [71]. The training process uses the Adam optimiser [72] to optimize the parameters, with a learning rate of 1e-3 and a weight decay of 5e-4. A fixed random seed of 1234 was used throughout the experiments to ensure reproducibility. To avoid overfitting of the network, the Dropout algorithm [73] is introduced during training. To control the training process, we employ the Early Stopping strategy [74], which is regulated by monitoring the model’s performance on the validation set, with a patience value of 10. In the model evaluation stage, a dynamic threshold optimization strategy [75] is adopted, and the threshold that maximises the F1 score is selected as the optimal decision boundary of the method. We select BCEWithLogitsLoss as the loss calculation function of the model [70].

### 4.4 Algorithm performance evaluation criteria

In this study, to comprehensively evaluate the model’s performance in the PepPI prediction task, we employed multiple evaluation metrics that reflect the model’s performance across different dimensions, including AUC, AUPR, Accuracy (ACC), and F1 score [76,77]. The calculation formulas for the relevant evaluation indicators are as follows [36]:


$$\begin{array}{c} \left\{ \begin{array}{cc} \text{ACC} & =\frac{TP+TN}{TP+TN+FP+FN}\\ \text{F1} & =\frac{\text{2}\times P\times R}{P+R} \end{array}\right.\; \end{array}$$ (6)

where TP represents the number of actual positive sequences, TN represents the number of true negative sequences, FP represents the number of false positive sequences, and FN represents the number of false negative sequences. Additionally, P denotes precision (the proportion of correctly predicted positive sequences among all predicted positive sequences) and R denotes recall (the proportion of correctly predicted positive sequences among all actual positive sequences). AUC is the area under the Receiver Operating Characteristic (ROC) curve, which plots the actual positive rate against the false positive rate at different classification thresholds. AUPR is the area under the Precision-Recall curve, which illustrates the relationship between precision and recall under varying threshold settings.

## Supporting information

S1 Table Performance comparison of PepLM-GNN with other baseline methods based on five-fold cross-validation. (DOCX)
