# Supplementary material for: PepLM-GNN: A graph neural network framework leveraging pre-trained language models for peptide-protein binding prediction
Source: PLoS Comput Biol. 2026 Mar 24;22(3):e1014084. doi: 10.1371/journal.pcbi.1014084 (PMC13012464; doi:10.1371/journal.pcbi.1014084)
Supplement: S1 Table — (DOCX) [file pcbi.1014084.s001.docx]

**S1 Table . Performance comparison of PepLM-GNN with other baseline methods based on five-fold cross-validation.**

| **Method** | **ACC** | **F1** | **AUC** | **AUPR** |
| --- | --- | --- | --- | --- |
| LR | 0.3082_±0.0063_ | 0.2937_±0.0080_ | 0.2444_±0.0075_ | 0.3557_±0.0024_ |
| RF | 0.3771_±0.0039_ | 0.4410_±0.0043_ | 0.3533_±0.0080_ | 0.4528_±0.0058_ |
| SVM | 0.5085_±0.0091_ | 0.5379_±0.0464_ | 0.5098_±0.0123_ | 0.5151_±0.0178_ |
| DrugBAN | 0.5044_±0.0062_ | 0.3971_±0.2664_ | 0.5187_±0.0074_ | 0.5133_±0.0071_ |
| HIGH-PPI | 0.5579_±0.0264_ | 0.6883_±0.0103_ | 0.6028_±0.0446_ | 0.5839_±0.0500_ |
| CAMP | 0.5739_±0.0911_ | 0.6831_±0.0229_ | 0.6001_±0.1226_ | 0.7342_±0.0219_ |
| IIDL-PepPI | 0.7481_±0.0033_ | 0.7511_±0.0113_ | 0.8269_±0.0035_ | 0.7956_±0.0087_ |
| **PepLM-GNN** | **0.7636_±0.0117_** | **0.7880_±0.0034_** | **0.8434_±0.0060_** | **0.8165_±0.0103_** |

**Note**: The results are presented as mean ± standard deviation. The best result for each metric ismarked in bold.
